# Short-term plasticity influences episodic memory recall: an interplay of synaptic traces in a spiking neural network model

**DOI:** 10.1038/s41598-025-12611-5

**Published:** 2025-08-01

**Authors:** N. Chrysanthidis, F. Fiebig, A. Lansner, P. Herman

**Affiliations:** 1 Division of Computational Science and Technology, School of Electrical Engineering and Computer Science, KTH Royal Institute of Technology, Stockholm, Sweden; 2 Department of Mathematics, Stockholm University, Stockholm, Sweden; 3 Digital Futures, KTH Royal Institute of Technology, Stockholm, Sweden; 4 International Research Center for Neurointelligence (IRCN), The University of Tokyo, Tokyo, Japan

**Keywords:** Episodic memory, Recency, Bayesian–Hebbian plasticity, Spiking cortical memory model, Attractor dynamics, Learning algorithms, Long-term memory, Short-term memory, Network models

## Abstract

We investigated the interaction of episodic memory processes with the short-term dynamics of recency effects. This work takes inspiration from a seminal experimental work involving an odor-in-context association task conducted on rats. In the experimental task, rats were presented with odor pairs in two arenas serving as old or new contexts for specific odor items. Rats were rewarded for selecting the odor that was new to the current context. These new-in-context odor items were deliberately presented with higher recency relative to old-in-context items, so that episodic memory was put in conflict with a short-term recency effect. To study our hypothesis about the major role of synaptic interplay of plasticity phenomena on different time-scales in explaining rats’ performance in such episodic memory tasks, we built a computational spiking neural network model consisting of two reciprocally connected networks that stored contextual and odor information as stable distributed memory patterns. We simulated the experimental task resulting in a dynamic context-item coupling between the two networks by means of Bayesian–Hebbian plasticity with eligibility traces to account for reward-based learning. We first reproduced quantitatively and explained mechanistically the findings of the experimental study, and then to further differentiate the impact of short-term plasticity we simulated an alternative task with old-in-context items presented with higher recency, thus synergistically confounding episodic memory with effects of recency. Our model predicted that higher recency of old-in-context items enhances episodic memory by boosting the activations of old-in-context items. We argue that the model offers a computational framework for studying behavioral implications of the synaptic underpinning of different memory effects in experimental episodic memory paradigms.

## Introduction

Episodic memory refers to an ability to recall past experiences. The uniqueness of these memories lies in their specific environmental context, as they are memorized in particular spatial locations at a given time^1^. Despite the multitude of past experiences, often sharing some contextual similarity, they can be vividly distinguished due to the specificity of the overall context with its episodic, typically both spatial and temporal, characteristics. Consequently, we can usually reliably order such long-term episodic memories in time^2,3^. It is less clear however how short-term plasticity, which transiently modulates synaptic efficacy, interacts with the encoding and recall of newly formed episodic memories. After all, the contextual binding that underlies episodic memory should be unique to specific events and experiences occurring in time. Our daily experiences are abundant in episodic memory recalls that involve an interplay of different memory processes across different time scales. It is not hard to conjure up a scenario where our very recent or even current experiences, often perceptual, affect the way we recall past events. For example, when we meet a person we may be somewhat confused about the episodic context, e.g. place, of our previous, and particularly recent, encounter, especially if it is different from the current circumstances. Temporal dynamics of recency has been extensively studied in the context of working memory^4–7^. Complementary computational work has further demonstrated that recency effects in working memory can be explained in recurrent network models by fast Hebbian^8^ or non-Hebbian (synaptic facilitation, depression) short-term plasticity^9,10^. There is evidence that most recently encountered task-relevant stimuli tend to have the highest likelihood of successful recall^11^. However, beyond the classical notion of working memory, newly formed intermediate-term (lasting longer than typical working memory maintenance and requiring higher memory capacity) episodic memories may also be subjected to short-term recency dynamics^12^. Overall, the influence of short-term recency dynamics in episodic memory recall is poorly understood. One of key challenges lies in experimental design providing scope for an effective manipulation of these short-term effects to gain deeper insights how they interact with episodic encoding giving rise to tangible behavioural outcomes. To date, the effect of recency, sometimes confounded with familiarity^13^, has been only sporadically examined in experimental studies concerned with episodic recall. From the computational perspective, most existing models simplify episodic memory in abstract, non‐spiking architectures with focus on systems level interactions attributing different roles to multiple subsystems, often disregarding mesoscopic mechanisms shaping the dynamics and function of the network’s neural substrate. A more detailed mechanistic framework accounting for biologically plausible neural and synaptic processes likely involved in immediate episodic recall is thus still lacking.

To acquire a better mesoscopic perspective by examining how synaptic‐ and circuit‐scale processes shape behavior (rather than systems level understanding i.e., role of distinct brain areas), we assumed that the same synaptic and neuronal substrate that supports newly formed episodic memories is also subject to fast short-term synaptic dynamics (milliseconds–seconds) as well as slower associative plasticity (tens of seconds). This mechanistic co‐localization enables us to study how these distinct synaptic processes at varying time scales jointly contribute to immediate recall, occurring shortly after the episodic encoding (contextual associative binding) is initiated. In particular, we used the same dual network model that was initially built to propose and assess a Bayesian–Hebbian hypothesis (synaptic changes based on co-activation probabilities, incorporating both pre- and postsynaptic activity in a probabilistic framework) about synaptic and network mechanisms underlying semantization of episodic memory, i.e. transformation of episodic memories to more abstract semantic representations^14^, a memory phenomenon documented in human studies^15,16^, and further observed in animal models^17^. With the intention to study functional implications of the aforementioned mesoscopic memory processes we were inspired by Panoz-Brown et al.’s^18^ study on episodic memory in rats. They devised a unique item-in-context task that helped them evaluate effects of fast recency biases in conjunction with slower memory processes of episodic encoding (item-context binding) on memory recall. To differentiate these effects they manipulated trial structure and odor presentation. In particular, Panoz-Brown et al.^18^ adapted an odor-span task involving a sequence of recently experienced odors to an episodic memory test with distinct environmental contexts—arenas where the odors were presented. Rats were rewarded for selectively responding to only those odors that were new to any given arena (new-in-context stimuli). To directly contrast the effects of recency and context-dependent (episodic) memory, new-in-context odors were typically presented more recently than odors previously encountered in the given context (old-in-context odors) prior to pairwise (“new” vs. “old”) odor memory assessment. The task was arranged so that rats had to overcome the short-term recency bias of new-in-context items and rely on an earlier episodic association between a given old-in-context odor and the contextual arena. Rats turned out to manage the task as they reliably performed episodic recall to claim reward even for retention intervals reaching 45 min. In our simulations we aimed at explaining the behavioral results reported by Panoz-Brown et al.^18^ as the emergent network effect of local synaptic plasticity at separate time scales. In particular, in line with the experimental study we concentrated on the temporal order aspects of recency dynamics and their impact on immediate episodic memory recall following short retention intervals. Additionally, we generated testable predictions for behavioral outcomes in modified experimental paradigms evaluating further interactions between episodic memory processes and recency dynamics. Importantly, to offer meaningful mesoscopic insights the model reflects a wide range of biological constraints and operates on behavioral time scales under constrained network connectivity with plausible postsynaptic potentials, spiking activities, and other biophysical parameters.

## Results

### A computational modelling approach to studying short-term memory phenomena in episodic memory recall

**Fig. 1:**
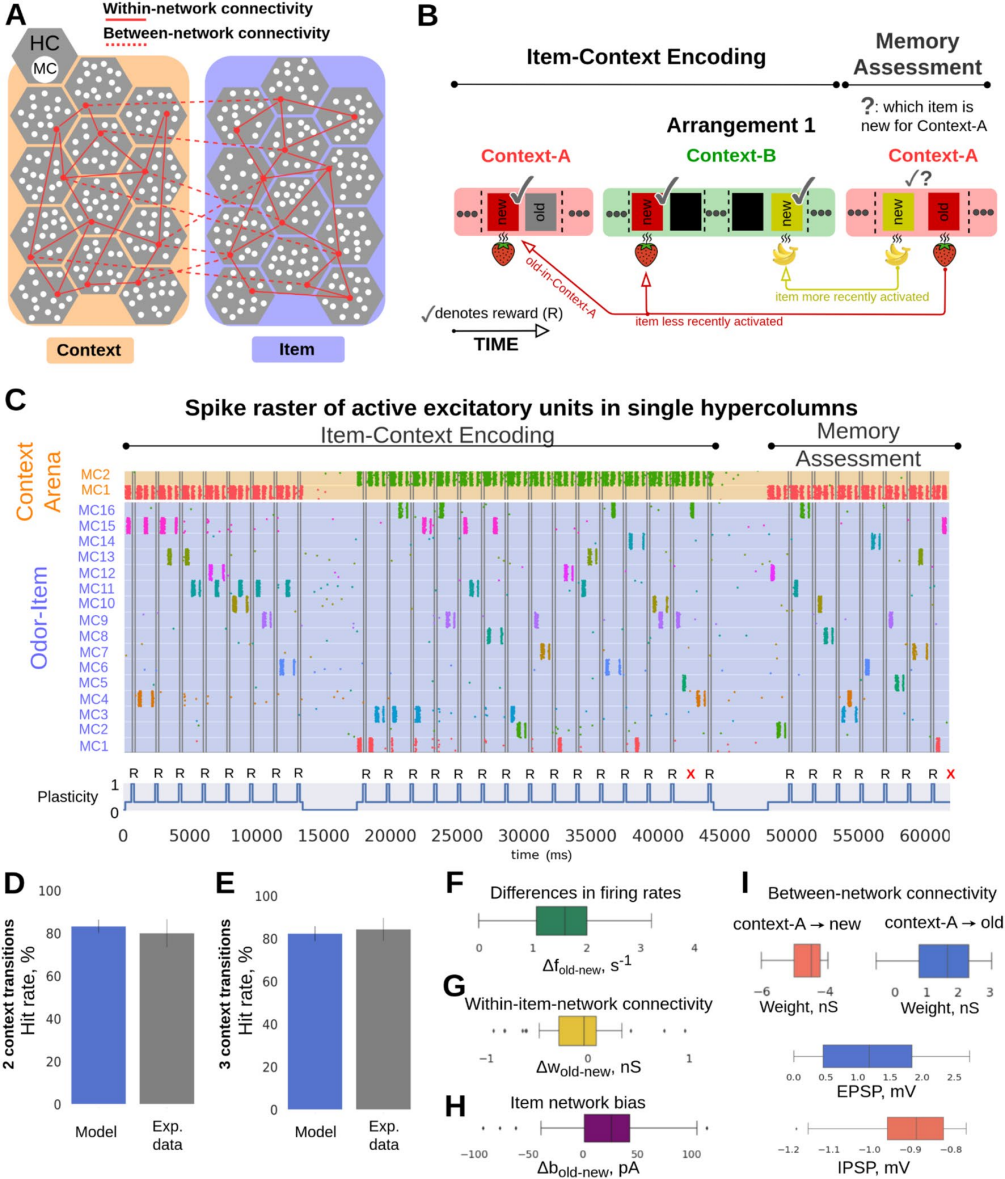
Item-in-context memory network model relying on associative episodic binding. (**A**) Graphic illustration of the Item (purple-blue) and Context (orange) networks. Attractor connections (solid red) represent within-network connectivity across hypercolumns (HCs) in the same network, while associative binding refers to the plastic connections between Item and Context networks (dashed red). (**B**) Task structure: odors were presented across two contexts in the simulated episodic memory task. Schematic of the two-context-transition task displaying pairs of new-old odors (depicted as rectangles with unique colors) in a given context [cf. Fig. 1B in Panoz-Brown et al.^18^]. Only the new items-in-context were rewarded (✓ symbol in the schematic denotes reward) when selected (a 100 ms stimulation of the selected odor preceded the reward phase, representing a final odor sniff before the reward). Once a new item was presented it was considered as old-in-context for the subsequent trials in the given context (as a trial we defined a stimulation of a pair of new- and old-in-context items). Items were stimulated for the first time in context-A, half of the total 16 items were presented and rewarded in context-A. After the context transition all the 16 items were presented in random pairs in context-B. Finally, Memory Assessment was made in context-A, where we presented the remaining half of the items that had not been presented in context-A (new-in-context-A items), and paired them randomly with old-in-context-A items (pairs of odors were different throughout the task). Context representations were constantly activated while cueing pairs of new-old items for 250 ms each. In the Memory Assessment block, pairs of new-old items followed the Arrangement 1 (new-in-context-A items were encoded more recently than old-in-context-A ones in the preceding context-B). Here, we show only 4 out of 16 items stimulated during the task (red and yellow items illustrate Arrangement 1). The presence of the additional items, which are not shown is indicated as “…”. For a simplified version of the item-in-context task, see Panoz-Brown et al.^18^. (**C**) Spike raster of pyramidal neurons in HC1 of the Item and Context networks simulating the episodic memory task described in (**B**). Item and context memory patterns are represented by the activation of a unique set of minicolumns (MCs) in their network. Each item or context was assigned with a unique color. While context representations were persistently cued we activated new and old items-in-context during trials. Plasticity of the associative binding between Item and Context networks was modulated during item presentation and rewarded accordingly (bottom subplot, R symbol in the schematic denotes reward, and X symbol, in red, indicates a failed trial). (**D,E**) The model discriminates between new- and old-in-context-A items with performance quantitatively matching Panoz-Brown et al.’s^18^ behavioral results in Experiment 1 (**D**) and Experiment 2 (**E**), respectively. Error bars represent SDs derived from the Bernoulli distributions for the probabilities of success (hit) across all trials (scaled to %), and for original experimental results—data is shown as mean + 1 SEM across rats. (**F**) Boxplot of the differences in average firing rates between pairs of old- vs. new-in-context-A items, Δf_old-new_. (**G**) Distribution of the differences in average within-network connectivity between pairs of old- vs. new-in-context-A items, Δw_old-new_, (within-network connectivity includes short-term plasticity mechanisms combined with intermediate-term Hebbian component: AMPA and slower NMDA receptor mediated weights). (**H**) Distribution of the differences in average intrinsic excitability between pairs of old- vs. new-in-context-A items, Δbias_old-new_. (**I**) Weight distribution of the episodic associative binding prior to the Memory Assessment part of the task. The distributions display the means of the learned synaptic weights (AMPA and slower NMDA receptor mediated weights, see Table 1) between context A and new-in-context-A items (blue, top right), or old-in-context-A items (red, top left). The figure also displays the excitatory (middle) and inhibitory (bottom) postsynaptic potentials (within biological plausible range^21^) of the corresponding weights distributions, which also account for the multiplicative effect of synaptic augmentation.

To address a general question of how episodic recall is subject to short-term memory phenomena at the level of network dynamics driven by synaptic plasticity mechanisms we employed a computational memory model consisting of two inter-connected spiking neural networks storing odor-items (16 odors) and context-arena memories (2 contexts), respectively, as distributed long-term (i.e. familiar) memory patterns. Before simulations of the experimental trial blocks, long-term item and context memory patterns were first embedded by means of prior Bayesian-Hebbian learning with multiple epochs and a long plasticity time constant. The resulting Hebbian within-network attractor projections (within-network connectivity, solid red lines, Fig. 1A) remained fixed throughout the simulated task. However, the effective synaptic efficacy can increase (via synaptic augmentation) transiently based on the recent history of each attractor memory activation.

During the actual computational memory task we simulated the process of encoding episodic memories in line with the experimental task design proposed by Panoz-Brown et al.^18^ as associative between-network connections binding odor and context memory items (between-network connectivity, dashed red lines in Fig. 1A) shaped by a range of synaptic plasticity effects e.g., Bayesian-Hebbian plasticity, integrating intrinsic excitability, (see “Methods”), and presynaptic short-term memory plasticity mechanisms of synaptic augmentation and depression reflecting recency dynamics, historically modelled in the working memory context^9,10^. Presynaptic short‐term dynamics of augmentation (or facilitation and depression can modulate synaptic gain on millisecond‐to‐second timescales based on recent activity, without any associative (Hebbian) weight change^19,20^. On-line contextual binding with fast synaptic plasticity was induced by reward as a rapid learning cue to account for the continual learning nature of this experimental paradigm. To that end, we employed eligibility traces in the framework of our Bayesian–Hebbian synaptic learning rule, which enable delayed reward learning through upregulated associative plasticity upon successful odor-in-context recall (see “Methods”). Eligibility traces refer to temporally extended markers of synaptic activity that enable delayed reward based learning when a reward is applied after 250 ms (see “Methods”). Finally, we simulated recall as a discriminative process between neural activity attributed to competing (old- vs. new-in-context) odor memory patterns presented as a pair of odor network stimuli with the simultaneous contextual cue active in the background. We hypothesized that the interplay of different synaptic plasticity mechanisms at different time scales is co-localized within the same network (rather than segregated in distinct brain areas), and thus reflected in the functional connectivity of the learned network (synaptic weights). To relate to the experimental outcomes reported by Panoz-Brown et al.^18^, we quantified the memory model’s performance based on the network activity (firing rates, see “Methods”).

#### Episodic memory vs. the recency effect: the control task design (arrangement 1)

We first used the model to simulate Panoz-Brown et al.’s^18^ base experimental setup, where rats were exposed to a rapid presentation of several odors across two arenas (A,B) serving as contexts for odor items (A → B → A, Experiment 1, Fig. 1B; Symbol “→” indicates a context transition). Our simulations followed the item-context association protocol adopted from the two-context-transition task denoted as “Experiment 1” in Panoz-Brown et al.’s^18^ study. It consisted of two main experimental blocks: Item-Context Encoding and Memory Assessment. In the first block, 8 odor items were presented in context-A, followed by all 16 odor items presented in context-B (one context transition, A → B). So half of the items presented in context-B were previously encoded in context-A. In the experiment, odors were always presented in pairs, new- vs. old-in-context items, and rats responded by selecting one of them as new, which marked an individual trial. In the model, we cued corresponding memory item patterns in the Item network in short succession (inter-stimulus period of 200 ms, see “Methods”) to simulate the serial process of first recognising one odor then the other in each pair (as a result of sniffing). Simultaneously, we cued the respective memory pattern in the Context network to account for contextual information, which resulted in cross-network binding of the cell assemblies representing odor items and contexts via associative Hebbian plasticity.

In the second block, referred to as Memory Assessment, every remaining odor (8 out of 16) that has not been presented in context-A, i.e. novel to context-A, was presented in a pair with another randomly selected odor that at the presentation time had already been encoded in that context, i.e. an old-in-context-A item. To reiterate, in this original task design proposed by Panoz-Brown et al.^18^ old-in-context-A items featured lower recency (Fig. 1B, Arrangement 1, old-in-context-A items were always presented earlier than new-in-context-A items prior to Memory Assessment block), so that correct retrieval of items had to entirely depend on the contextual association. Recency might increase the sense of familiarity of an item, thereby potentially confusing rats. Therefore, in that arrangement, context-dependent episodic memory recall was put into conflict with the effect of memory recency (Fig. 1B, Arrangement 1). Old- and new-in-context-A items were cued only once during the Memory Assessment block. This is in contrast with the aforementioned Item-Context Encoding block where different items could be repeated multiple times and thus old-in-context items had always higher recency compared to new-in-context items (here: old/new relative to the given context A or B, in the Item-Context Encoding block) as the old-in-context items were encoded in the same context before the new-in-context item was presented. Odor pairs were different between Item-Context Encoding block and Memory Assessment, and also randomized across simulations. Accordingly, behavioral data in the experimental study and simulated data of the model performance here were examined only during the Memory Assessment block.

In Fig. 1C we illustrate an exemplary spike raster of active pyramidal neurons in one of the network hypercolumns (see “Methods”) of both the Item and the Context network obtained in a simulation of the entire experimental session. The bottom of Fig. 1C depicts the associative plasticity gain (item-context binding) modulation. It accounts for a reward signal (Fig. 1C, “R”: reward) that in line with the original experiment follows each successful odor choice (a continual learning scenario). The reward implementation uses synaptic eligibility traces and temporarily boosts associative plasticity gain from the baseline level, κ_normal_ (Table 1), during item presentation to the elevated κ_reward_ (Table 1). The odor choice (old- vs. new-in-context) itself was made based on a comparison between average firing rates elicited by the excitatory units corresponding to the two stimulated (competing) item patterns in each pair (see “Methods”).

By tuning stimulus-related parameters (i.e., strength of simulations and background noise excitation) of our earlier model on item-context episodic memory binding^14^, we obtained task performance comparable to the original experimental data (Fig. 1D,E; model data: mean = 83.21, SD = 3.12, n = 143, mean represents the total number of successes across all n-trials [each trial tests one old-new pair], SD derived from the Bernoulli distributions for the probabilities of successes across all n-trials, n corresponds to simulated old-new pairs during Memory Assessment, and experimental data: mean≈80, SD≈6.5, mean reflects the averaged performance of rats in 9 sessions, combining the initial and terminal sessions, and the error bars reflect the averaged standard error of the mean (SEM) across rats for the combined initial and terminal sessions) for the two-context-transition task (Experiment 1, see Ref.^18^). The high odor item recall performance of the model originates from considerably stronger network response elicited on average by old- relative to new-in-context items (Fig. 1F, differences between averages in firing rates induced by pairs of old- vs. new-in-context-A items, Δf_old-new,_ are positive, and hence old-in-context-A items elicited stronger response, see Pairwise differences section in “Methods”). To gain further insights in ways inaccessible to in-vivo experiments, we examined the synaptic strength of the within- and between-network connectivity, and neuronal excitability dynamics (BCPNN bias, see “Methods”). The high odor recall performance cannot be explained by observations in the within-network connectivity, as the differences in average within-network connectivity between pairs of old- vs. new-in-context-A items, Δw_old-new_, drifts towards negative values (Fig. 1G, see Pairwise differences section in “Methods”), primarily due to high recency of new-in-context-A items which boosts their connectivity. Regarding the bias factor, old-in-context-A items were typically presented more times than new-in-context-A items, as a consequence of task design, the cell assemblies corresponding to more repetitive old-in-context-A items exhibited higher neuronal excitability. The distribution of the differences in average bias between pairs of old- vs. new-in-context-A items, Δb_old-new_, is positive, and favors old-in-context-A items in Fig. 1H, which partly contributed to the odor recall performance. Prior to the Memory Assessment block, items that were presented in context-A established an excitatory associative binding (Fig. 1I, top right, EPSPs in middle) unlike other items, never cued in context-A beforehand, were subjected to disynaptic inhibition (Fig. 1I, top left, IPSPs in bottom, see Methods for the disynaptic inhibition implementation in our model). During the Memory Assessment block, which was still part of the continual learning process in context-A, items that were initially new to that context became old-in-context-A items once they were used as a stimulus in the Memory Assessment. Hence, plastic disynaptic inhibition built during the earlier Item-Context Encoding block was transformed to excitatory binding (continual learning process) after the odor item was cued in the Memory Assessment block (see Methods). All in all, the synaptic weights of the associative item-context binding and the bias factor contributed to the observed difference in firing rates between old- vs. new-in-context-A items in Fig. 1F, counteracting the effect of recency dynamics of new-in-context-A items.

Next we challenged our model by simulating the extended task with three context transitions (A → B → A → Memory Assessment block in context B), as proposed by Panoz-Brown et al.^18^. In their second experiment (denoted as Experiment 2), 8 out of 16 odors were stimulated in context-A (as before in Experiment 1), followed by 8 odors in context-B. After transitioning to context-A again, the remaining 8 items not shown in context-A yet were presented. The Memory Assessment part of Experiment 2 was then conducted in context-B, unlike in Experiment 1, with new-in-context-B items presented along with the previously encoded old-in-context-B items, as before (Fig. S1, an example of a three-context-transition task). The same model, i.e. without any further re-tuning, reproduced again quantitatively similar odor recall performance as in Panoz-Brown et al.’s^18^ Experiment 2 (Fig. 1E; model data: mean = 82.35, SD = 3.49, n = 119, mean represents the total number of successes across all n-trials, SD derived from the Bernoulli distributions for the probabilities of successes across all n-trials, n corresponds to simulated old-new pairs, and experimental data: mean≈84, SD≈5.3, mean reflects the average performance of rats in 9 sessions, combining the initial and terminal sessions, and error bars reflect the average standard error of the mean (SEM) across rats for the combined initial and terminal sessions).

#### Synergy of episodic memory and recency: an alternative task design (arrangement 2)

**Fig. 2:**
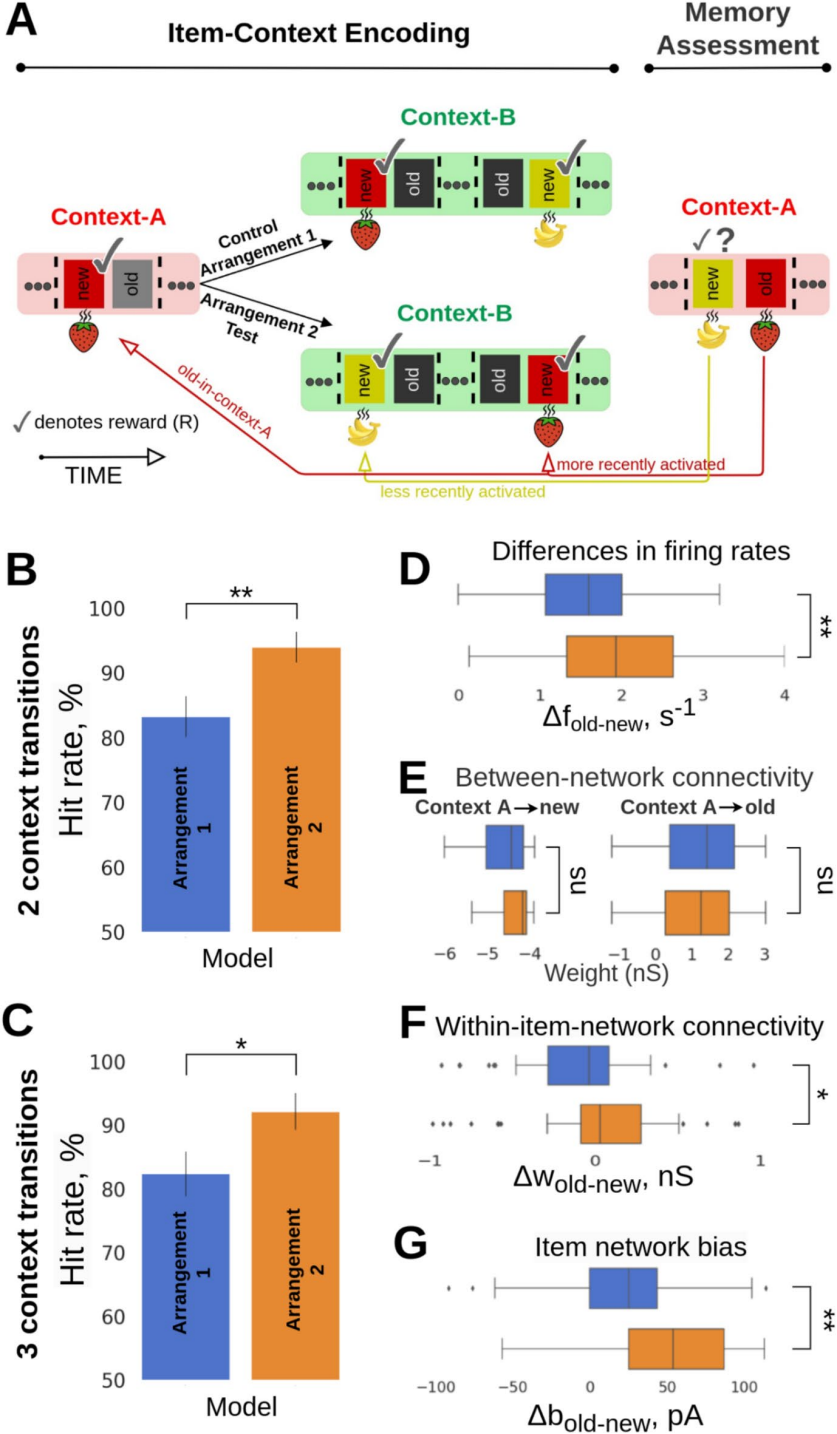
(**A**) Structure of the episodic memory task: Arrangement 1 vs. Arrangement 2. In Arrangement 1 (cf. Fig. 1B), the new-in-context-A item was encoded more recently than the old-in-context-A item prior to the Memory Assessment phase. This order was reversed in Arrangement 2. (**B**,**C**) Model performance in new- vs. old-in-context memory discrimination between Arrangements 1 and 2 for two- and three-context transitions, respectively. Error bars represent SDs derived from the Bernoulli distributions for the probabilities of success (hit) across all trials (scaled to %). (**D**) Boxplot of the differences in average firing rates between pairs of old- vs. new-in-context-A items, Δf_old-new_, in Arrangement 2 (bottom) and in Arrangement 1 (top). The trial-average firing rates represent the means of evoked spiking frequency during the activation of a given item during a test trial in the Memory Assessment phase. (**E**) Synaptic connectivity (AMPA and slower NMDA receptor mediated weights) between Item and Context networks is similar in both arrangements and remains resistant to changes (i.e., order of item activation) due to encoding with intermediate-term Hebbian plasticity (τ_p_ = 30 s, Table 1). (**F**) Distribution of the differences in average within-network connectivity between pairs of old- vs. new-in-context-A items, Δw_old-new_, for the Arrangement 1 (blue) and Arrangement 2 (orange). (**G**) Distribution of the differences in average intrinsic excitability between pairs of old- vs. new-in-context-A items, Δbias_old-new_, for the Arrangement 1 (blue) and Arrangement 2 (orange). Both within-network connectivity and bias effects of old-in-context-A items within the Item network are stronger in Arrangement 2 due to recency, thus leading to higher performance as observed in B.

To prevent rats from utilizing any semantic rules concerned with items’ recency, Panoz-Brown et al.^18^ randomly intermingled Arrangement 1 trial blocks with trials of an alternative structure called Arrangement 2. In Arrangement 2, the order of the item presentation in the Item-Context Encoding block prior to the Memory Assessment was switched so that old-in-context-A items featured higher recency than the new-in-context-A items (Fig. 2A, Arrangement 1 vs. Arrangement 2). Panoz-Brown et al.^18^ did not report any experimental results for Arrangement 2 as the recency effect might be confounded with context-dependent episodic memory. We nevertheless wanted to provide a qualitative prediction about the memory performance in the Arrangement 2 task given the synergy of short-term recency and episodic memory. It is worth noting that the number of trials simulated for Arrangements 1 and 2 were slightly different as the sampling of odor pairs was subject to different constraints regarding their new vs old status across the contexts.

As expected, the model predicted higher odor recall performance in Arrangement 2 than in Arrangement 1 for both two- (Fig. 2B, Arrangement 1: mean = 83.21, SD = 3.12, n = 143 vs. Arrangement 2: mean = 93.33, SD = 2.39, n = 99; p < 0.01, Fisher’s exact test), and three-context transitions (Fig. 2C, Arrangement 1: mean = 82.35, SD = 3.49, n = 119 vs. Arrangement 2: mean = 92.13, SD = 2.85, n = 89; p < 0.05, Fisher’s exact test). To elucidate the synaptic origins and network correlates of the performance enhancement, we analyzed key model variables such as spiking activity of excitatory units representing the old- and new-in-context items, synaptic strength of the within- and between-network connectivity, and neuronal excitability dynamics (BCPNN bias, see “Methods”). We observed that the differences between the averages in firing rates induced by old- vs. new-in-context-A items, Δf_old-new,_ increased significantly in Arrangement 2 relative Arrangement 1 (Fig. 2D; p < 0.01, two-sample t-test, 90 simulated trials [pairs of odors in the Memory Assessment] in Arrangement 1 and 62 in Arrangement 2, see Pairwise differences section in Methods), implying the improved capability of the model to discriminate and accurately identify new-in-context-A odor items. We partially attributed this to the temporary enhancement in the strength of the within-network connectivity (Fig. 2F, p < 0.05, Mann–Whitney U test, 47 simulated trials [pairs] in Arrangement 1 and 41 trials in Arrangement 2). As mentioned earlier, the within-network connectivity was preloaded (long-term memory representations of items and contexts were encoded prior to the Item-Context Encoding block), so it was short-term synaptic augmentation that rapidly upregulated the effective synaptic weights. This enhancement was short-lasting, limited by the augmentation time constant, and thus it could only be effective when the stimulation of a given item in context-B was within a narrow time window relative to the temporal scales of the Memory Assessment block in context-A. Furthermore, we observed a notable increase in the difference between the neuronal excitability (Δbias) for old- vs. new-in-context-A items in Arrangement 2 (Fig. 2G, p < 0.01, Mann–Whitney U test, 47 simulated trials [pairs] in Arrangement 1 and 41 trials in Arrangement 2). Old-in-context-A items were stimulated more often than the new-in-context-A ones as a result of the altered task structure (Fig. 2A). In Arrangement 2 the final stimulation of an old-in-context-A item in the preceding context-B had to take place after the most recent activation of a new-in-context-A item even if there were cases that old-in-context-A items had been activated before (i.e., strawberry [“old-in-context-A”, first stimulation in “context B”]–banana [“new-in-context-A”, first stimulation in “context B”]–strawberry [“old-in-context-A”, second stimulation in “context B”]). Enforcing Arrangement 2 therefore enhanced the intrinsic neural excitability of old-in-context-A items. It is worth mentioning that modification of the temporal order of items between Arrangement 1 and Arrangement 2 did not yield any meaningful change for the between-network connectivity (Fig. 2E, p > 0.05, Mann–Whitney U, 47 simulated trials [pairs] in Arrangement 1 and 41 trials in Arrangement 2). The between-network connectivity was long lasting ($\tau$_p_ = 30 s, Table 1), as before, to support episodic retrieval, i.e. to continuously perform an item-context association task spanning many consecutive trials. As a digression, we observed in our unpublished preliminary study that fast Hebbian plasticity of the between-network connectivity with short time synaptic constants (e.g., with a time constant $\tau$_p_ of 5 s corresponding to working memory settings by other models) could not solve the task, as the temporal memory traces of previously encoded item-context pairs decayed rapidly from trial to trial.

#### Unbalanced training paradigm with two context transitions

**Fig. 3:**
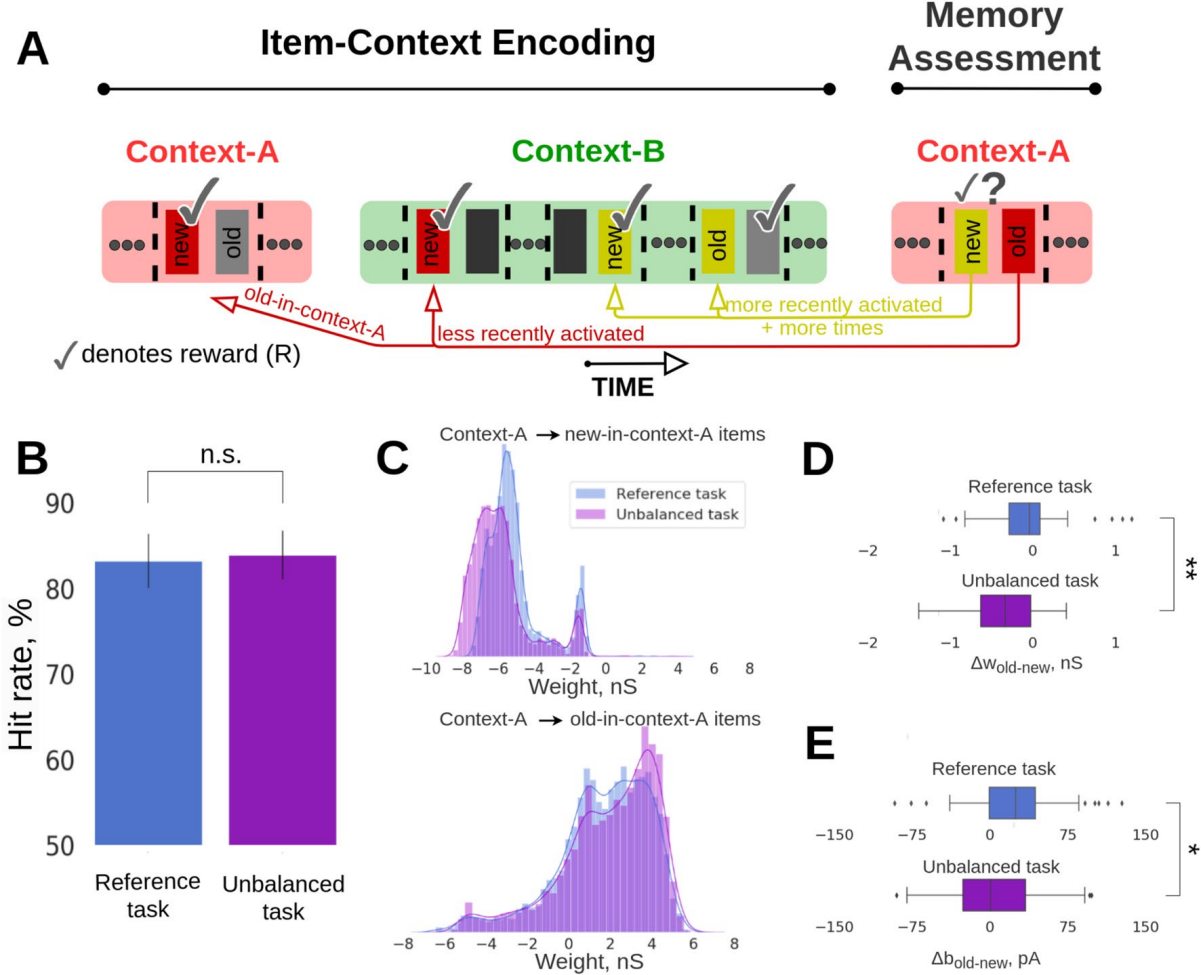
Unbalanced training prediction task. (**A**) Schematic of the unbalanced training task. As in the reference two-context-transition task (Fig. 1B), half of the items were presented in context-A, followed by the presentation of all the items in context-B. The Memory Assessment was conducted in context-A by presenting pairs of new-old items. However, for the unbalanced training task, we stimulated more times the new-in-context-A items in the preceding context-B than in the corresponding reference task. (**B**) Average recall performance (hit rate, %) for the reference and unbalanced prediction task corresponding to Arrangement 1 configuration. SDs derived from the Bernoulli distributions for the probabilities of success (hit) across all trials (scaled to %). (**C**) Distributions of associative weights (AMPA and slower NMDA receptor mediated weights, reported prior to the Memory Assessment phase) between context-A and new-in-context-A items (top, disynaptic inhibitory weights), and between context-A and old-in-context-A items (bottom) for the reference and unbalanced training prediction task. (**D**) Boxplot of the differences in average within-network connectivity between old- vs. new-in-context-A items, Δw_old-new_, for the reference task with two context transitions (top) and the unbalanced training scenario (bottom). (**E**) Boxplot of the differences in average intrinsic excitability (neuronal bias) between pairs of old- vs. new-in-context-A items, Δb_old-new_, for the reference task with two context transitions and the unbalanced training scenario.

To further exploit the predictive capabilities of the model, we examined the effect of the frequency of stimulus presentation (multiplicity or repetition of stimuli) as a potential factor modulating the item familiarity on the item-in-context recall. In particular, we set out to study if the stimulus multiplicity on top of the recency would synergistically outcompete the episodic memory effect in the old- vs. new-in-context item choice, thereby leading to the higher recall error rate. To this end, we resorted to Arrangement 1 (competition between recency and episodic memory phenomena). However, unlike in the balanced Experiment 1, we now increased the number of new-in-context-A odor presentations resulting in an unbalanced scenario where new-in-context-A items were presented more frequently (i.e., in Fig. 3A in the Memory Assessment block, the new-in-context-A item (yellow) had been used twice in the preceding context-B, while the old-in-context-A item (red) had appeared only once in the preceding context-B). Surprisingly, our expectation that the enhanced familiarity due to increased multiplicity along with recency should outcompete episodic memory and “mislead” the model in the old- vs. new-in-context-A choice during the Memory Assessment turned out to be false. In fact, we found evidence of comparably high performance for the unbalanced training task, i.e. reference task: mean = 83.21, SD = 3.12, n = 143 vs. unbalanced training task: mean = 83.92, SD = 2.83, n = 168; p > 0.05, Fisher’s exact test.

We next sought to mechanistically explain the comparable performance in the unbalanced task, which was opposite to our expectation as we predicted lower performance. First, we analyzed the between-network connectivity and found that disynaptic inhibition between context-A and the new-in-context-A items was strengthened (Fig. 3C, top, p = 1.9 × 10^–268^, Mann–Whitney U-test, 6638 weights from Context-A to all the new-in-context-A items for the reference task vs. 5382 weights from Context-A to all the new-in-context-A items for the unbalanced task). This effectively resulted in a more negative (inhibitory) association in the unbalanced training task. There were more opportunities for new-in-context-A items to be repeated in context-B of the Item-Context Encoding block than in the reference task setup. This strengthened not only their associative binding with context-B but also their dissociation with context-A (mediated by plastic disynaptic inhibition, see Methods for the disynaptic inhibition implementation in our model). At the same time, associative excitatory binding between context-A and those items that were later considered old-in-context-A in the Memory Assessment block became stronger, predominantly due to their less frequent presentation as a stimulus in the competing context-B. Indeed, in the spirit of Bayesian nature of BCPNN learning, a more specific and exclusive pairing of two memory patterns induces stronger associative binding. The observed changes of the between-network connectivity (Fig. 3C, bottom, p = 7.82 × 10^–16^, Mann–Whitney U-test, 6725 weights from Context-A to all the old-in-context-A items for the reference task vs. 5464 weights from Context-A to all the old-in-context-A items for the unbalanced task) can explain the reason why the hit rates were not reduced for the unbalanced training task (Fig. 3B). However, differences in the within-network connectivity and bias still hurt recall, because new-in-context-A items featured stronger attractor connectivity (Fig. 3D, enhanced within-network connectivity of new-in-context-A items in the unbalanced task drove the Δw_old-new_ distribution towards more negative values, reference vs. unbalanced task: p < 0.01, Mann–Whitney U-test, n_Reference_ = 47, n_Unbalanced_ = 59, see Pairwise differences section in Methods), and boosted neuronal excitability (bias, Fig. 3E, references vs. unbalanced task: p < 0.05, Mann–Whitney U-test, n_Reference_ = 47, n_Unbalanced_ = 59) compared to the original task design due to the additional stimulus repetitions. These effects led to a stronger competition between recency and episodic memory effects compared to the reference task. Still, our simulations showed that episodic memory processes reflected in associative between-network binding overpowered familiarity and recency effects manifested at the item representation level.

Collectively, the alterations in context-item binding, reflected in the between-network connectivity weights and caused by varying multiplicity of item presentations, resulted in maintaining a comparable high item-in-context memory performance. Due to the important role of the aforementioned disynaptic inhibition between context-A and new-in-context-A items, we conducted a follow-up experimental manipulation by severing the disynaptic weights connecting the networks for the unbalanced task. Subsequent recall rates in the Memory Assessment block showed a dramatic decrease in performance (Fig. S2, unbalanced task: mean = 83.92, SD = 2.83, n = 168 vs. “No disynaptic inhibition” task: mean = 16.66, SD = 5.37, n = 48; p < 0.001, Fisher’s exact test). Similar manipulation (e.g., removal of between-network disynaptic inhibitory weights) to the reference task leads to poor performance as well.

#### Memory interference by an additional episodic context

**Fig. 4:**
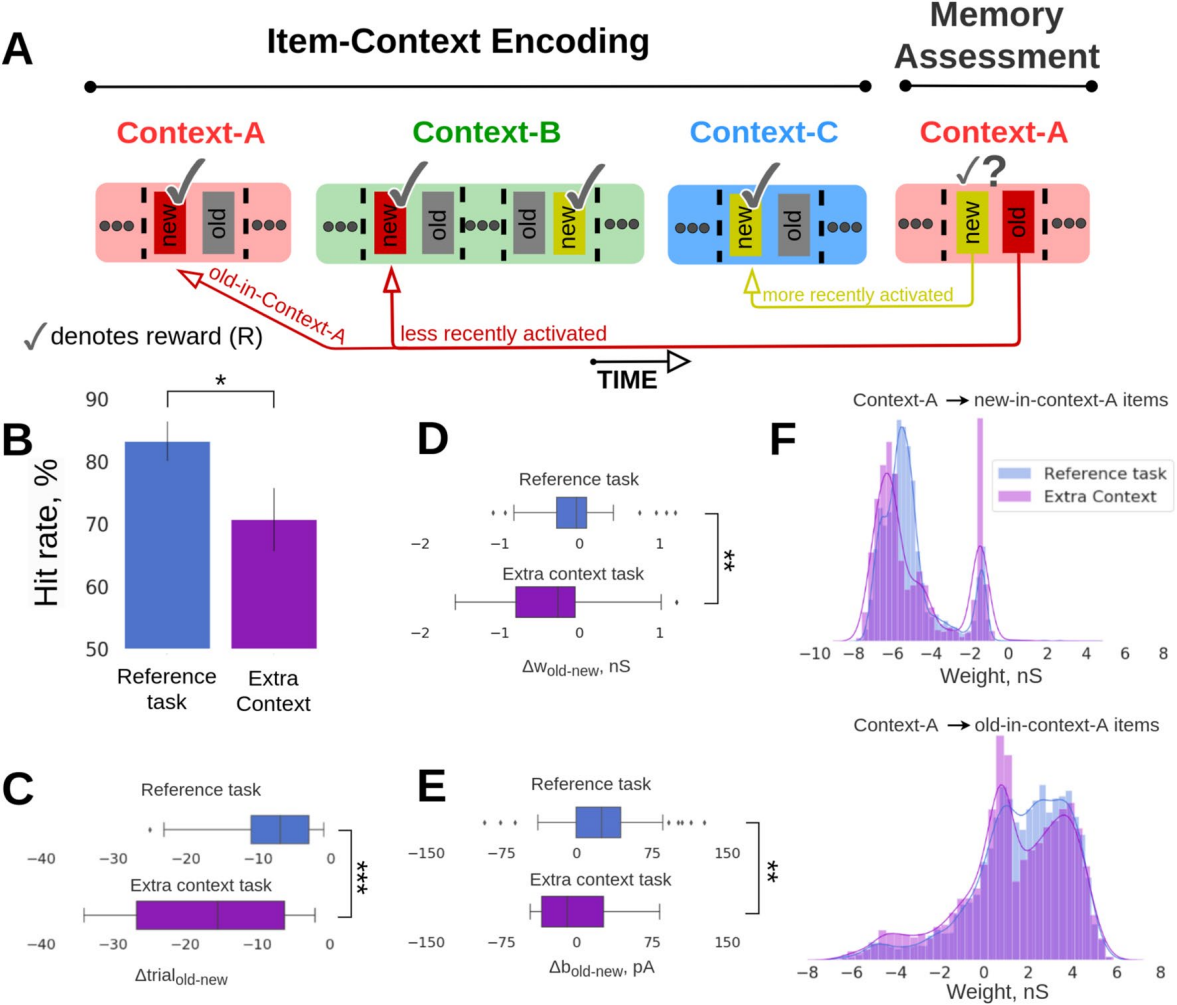
Extra context prediction task. (**A**) Schematic of the extra context task. Introducing an extra context (blue) in the Item-Coding Encoding block prior to the Memory Assessment block, in which we simulated randomly half of the 16 items in new-old item pairs. (**B**) Average recall performance (hit rate, %) for the reference and extra context prediction tasks corresponding to Arrangement 1 configuration. SDs derived from the Bernoulli distributions for the probabilities of success (hit) across all trials (scaled to %). (**C**) Boxplot of the differences in average trial-index between pairs of old- vs. new-in-context-A items, Δtrial_old-new_, for the reference task with two context transitions (top) and the extra context task (bottom). As a trial-index we define the trial index of the most recent activation of the items (i.e., a new-in-context-A item with trial-index = 40 is more recently encoded compared to an old-in-context-A item with trial-index = 30, and their relative recency difference is Δtrial_old-new_ = − 10). When an item was activated at the very first trial block, it was assigned with a trial-index = 1, and once it was activated again at a later trial, the trial-index was flexibly updated to correspond to the last activation position. The figure shows that the difference in trial-index between old- and new-in-context-A items becomes more negative for the extra context task, thus increasing their relative recency. (**D**) Boxplot of the differences in average within-network connectivity between pairs of old- vs. new-in-context-A items, Δw_old-new_, for the reference task with two context transitions (top) and the extra context task (bottom). (**E**) Boxplot of the differences in average intrinsic excitability (neuronal bias) between pairs of old- vs. new-in-context-A items, Δbias_old-new_, for the reference task with two context transitions and the extra context scenario. (**F**) Distributions of associative weights (reported prior to the Memory Assessment block) between context-A and new-in-context-A items (top, disynaptic inhibitory weights), and context-A and old-in-context-A items (bottom) for the reference and extra context tasks.

From the behavioral perspective on episodic memory it is interesting to study the effect of memory interference by introducing yet another context-C (arena) just preceding the Memory Assessment block (Fig. 4A). Increasing the complexity of the task (extended memory and temporal demands) by introducing additional contexts is a method often used in item-in-context episodic memory tasks on rats, and this process typically leads to lower performance scores^22^. Our intention was to make behaviourally relevant predictions about the odor recall performance in a more challenging setup compared to the reference task, and quantify potential behavioral changes in performance. In line with previous related behavioral experiments, the recall performance in our extra context task dropped significantly compared to the reference task (Fig. 4B, reference task: mean = 83.21, SD = 3.12, n = 143 vs. extra context task: mean = 70.7, SD = 5.02, n = 82; p < 0.05, Fisher’s exact test).

The new simulated task puts short-term recency effect in conflict with episodic memory, just as in the behavioral task with Arrangement 1, though in a more complex and longer item-in-context configuration facilitated by an extra context-C. In particular for this new context-C, we cued randomly 8 of the available 16 memory items (see Fig. 4A) following an analogous procedure of presenting items as in Experiment 1 (i.e., Fig. 1C, 0–15 s). Later, items that had been cued in context-C, could be used in the Memory Assessment block. Given the Arrangement 1 criteria (“new-in-context-A items should be more recently encoded in the preceding contexts than the old-in-context-A items”), we observed that new-in-context-A items (from the perspective of Memory Assessment) were the items that were mainly activated in the extra context-C (latest presentation before the Memory Assessment block) as opposed to the old-in-context-A items whose most recent presentation took place in context-B (not in context-C, Fig. 4A). This was an emerging outcome of the new task setup combined with Arrangement 1 requirements. Since old-in-context-A items were rather infrequent in trials belonging to context-C as opposed to new-in-context-A items that were activated extra times in context-C, there was a longer temporal distance between the trials of the most recent activation of a new- and its old-in-context-A item pair (higher relative recency between pairs of items, Fig. 4C, reference vs. extra context task: p < 0.001, Mann–Whitney U test, n_Reference_ = 47, n_ExtraContext_ = 34). The longer emerging temporal distance (higher relative recency) between old- and new-in-context-A items, led to enhanced within-network connectivity for new-in-context-A items (Fig. 4D, Δw_old-new_ distribution drifts to more negative values indicating within-network connectivity enhancement of new items, reference vs. extra context task: p < 0.01, Mann–Whitney U-test, n_Reference_ = 47, n_ExtraContext_ = 34, see Pairwise differences section in Methods). Also, the extra activations of new-in-context-A items in context-C yielded stronger learned intrinsic excitability (higher multiplicity hypothesized to enhance familiarity in experimental memory settings) compared to the reference task (Fig. 4E, Δb_old-new_ distribution drifts to negative values, reference vs. extra context task: p < 0.01, Mann–Whitney U-test, n_Reference_ = 47, n_ExtraContext_ = 34). The above changes in within-network connectivity and intrinsic excitability boosted spiking activity of new-in-context-A items making it harder for the model to distinguish between new- and old-in-context-A items (selection was made based on significant spiking activities differences between old- and new-in-context-A items), and hence these synaptic- and neuronal-level changes may explain the observed performance decline. Last but not least, we observed a similar disynaptic inhibition trend for the extra context task as in the previous unbalanced training task (Fig. 3C, top), that is, stronger disynaptic inhibition from context-A to all the new-in-context-A items compared to the reference task (Fig. 4F, top, reference vs. extra context task: p = 5.32 × 10^–8^, Mann–Whitney U-test, n_Reference_ = 6638 weights, n_ExtraContext_ = 5402 weights). However, the between-network connectivity (associative episodic item-in-context binding) was weaker compared to the reference task. Even though old-in-context-A items were activated infrequently in the extra context-C, still they were activated more times in other contexts compared to the reference task, and hence this additional repetition in other contexts can weaken the associative binding as shown in Chrysanthidis et al.^14^ study (Fig. 4F, bottom, reference vs. extra context task: p = 4.7 × 10^–13^, Mann–Whitney U-test, n_Reference_ = 6725 weights from context-A to all the old-in-context-A items, n_ExtraContext_ = 5453 weights from context-A to all the old-in-context-A items). It is worth noting that the majority of old-in-context-A items that were cued in context-C followed the stimulation logic of Arrangement 2 (Fig. 2A), i.e. old-in-context-A items were more recently encoded than the new ones, and were excluded from the Arrangement 1 analysis.

#### Reverse rewarding scheme

**Fig. 5:**
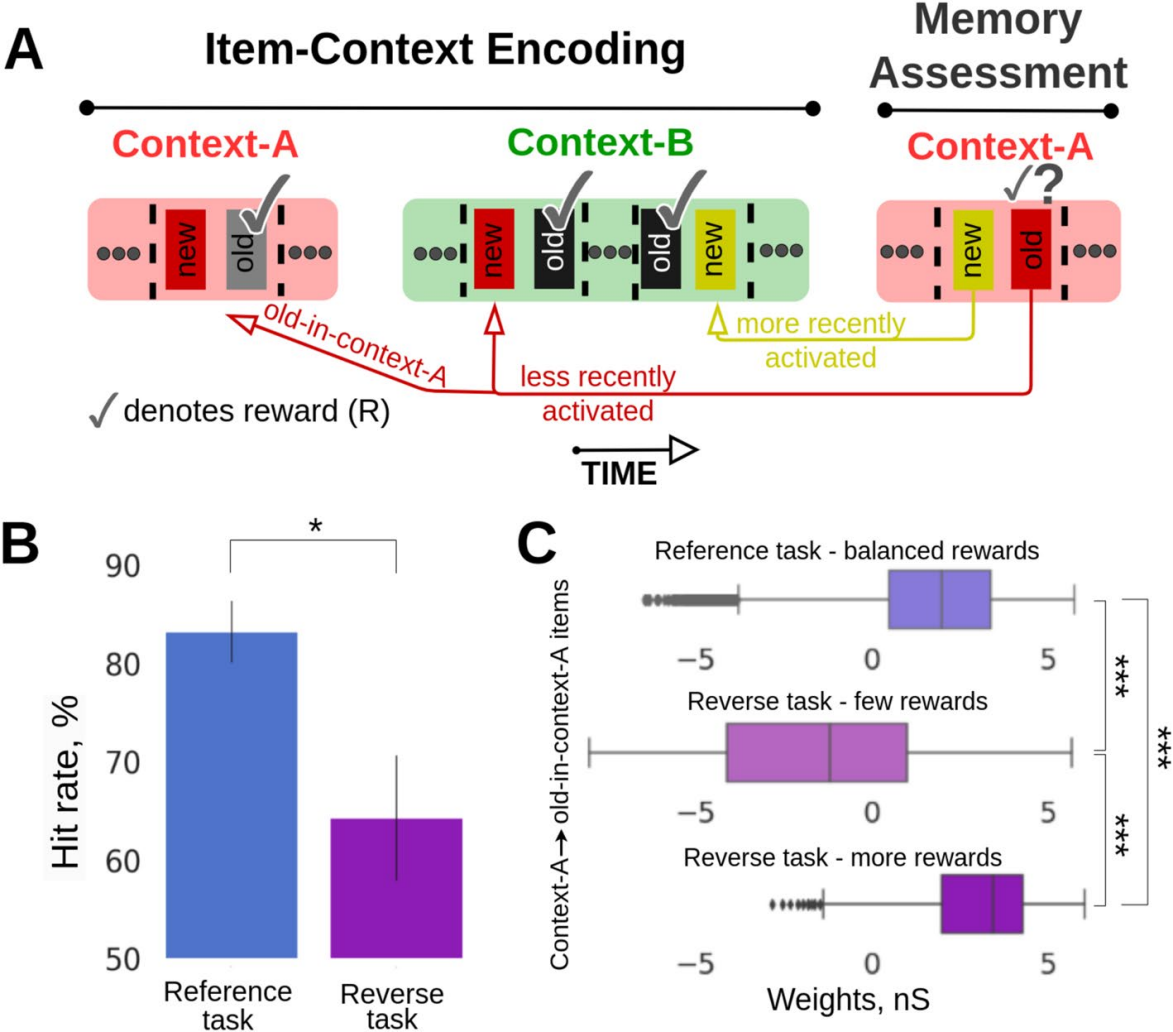
Reverse rewarding prediction task. (**A**) Schematic of the reverse rewarding scheme which was similar to the reference task with the only difference that old-in-context-A items were rewarded instead of new-in-context items. (**B**) Average recall performance for the suite of the prediction task. Data is shown for Arrangement 1. SDs derived from the Bernoulli distributions for the probabilities of success (hit) across all trials (scaled to %). (**C**) Boxplots of the differences in the between-network connectivity (associative weights, AMPA and slower NMDA receptor mediated weights, reported prior to the Memory Assessment block) between context-A and old-in-context-A items for the reference task (top), and between context-A and old-in-context-A items that were rewarded few times during the reverse rewarding scheme (middle), and between context-A and old-in-context-A items that were rewarded multiple times during the reverse rewarding scheme (bottom). Old-in-context-A items that were rewarded multiple times (i.e., more than two rewards) in the reverse rewarding scheme featured stronger synaptic connectivity with context-A (bottom) than the corresponding ones rewarded fewer times in the reverse rewarding scheme (middle).

In the original reference task (Fig. 1B) new-in-context items were rewarded only once upon selection since after the reward they were treated as old-in-context items, and no further reward was provided even after another presentation in the same context. Therefore, the overall reward was distributed uniformly across odors in both contexts in the Item-Context Encoding prior to the Memory Assessment block. To diversify reward distribution and study the impact of reward imbalance between items on memory recall, we reversed the reward scheme and changed the rule to provide rewards only to old-in-context items. Under this new rule, an old-in-context item could be rewarded as many times it was activated in the context (Fig. 5A). While most episodic memory tasks build on rewarding behavioural responses to novelty, there are still some experimental studies, particularly in rodents, where the recall of older memories is treated preferentially and rewarded^23^, in line with our “reverse rewarding” rule.

The reverse rewarding scheme substantially hurt model performance (Fig. 5B, reference task: mean = 83.21, SD = 3.12, n = 143 vs. reverse rewarding scheme: mean = 64.28, SD = 6.4, n = 56; p < 0.05, Fisher’s exact test). The poor memory performance is best explained by the between-network connectivity, which affects item-in-context memory performance, as observed in earlier prediction tasks. The reward imbalance resulted in varying levels of between-network connectivity strength among old-in-context-A items, promoting less robust associative episodic memory binding for some old-in-context-A items (Fig. 5C, difference between the means of associative weights between reference task and reverse task; cases with more and few odor rewards, i.e. reference: reward balance, vs. reverse rewarding scheme: reward imbalance; more rewards, p < 0.001, Mann–Whitney U test, n_Reference_ = 6725, n_more-Rewards_ = 2154, n represents the weights from context-A to all the old-in-context-A items; reference: reward balance, vs. reverse rewarding scheme: reward imbalance; few rewards, p < 0.001, Mann–Whitney U test, n_Reference_ = 6725, n_few-Rewards_ = 712; and reverse rewarding scheme: reward imbalance; more rewards, vs. reverse rewarding scheme: reward imbalance; few rewards, p < 0.001, Mann–Whitney U test, n_more-Rewards_ = 2154, n_few-Rewards_ = 712.

Weak between-network connectivity for old-in-context-A items that were rewarded fewer times decoupled the Item-Context networks (Fig. 5C). On the other hand, when old-in-context-A items were rewarded multiple times, their between-network connectivity was strengthened at the cost of other coupled items (in context-A), because learning was continuous throughout the task. Bayesian learning normalizes and updates weights continuously over estimated presynaptic (Bayesian-prior) as well as postsynaptic (Bayesian-posterior) spiking activity. We noticed that the reward imbalance in the reverse rewarding scheme, particularly for these cases where old-in-context-A items were rewarded less often led to incorrect odor choices. On the whole, we note that reward imbalance, rather than simply the reversal of the readout, accounts for the poorer performance under the reverse rewarding scheme training.

## Discussion

Testing episodic memory is important to elucidate the mechanisms that could interfere with, enhance or impair this memory system. While there is a wealth of research on behavioral manifestations of this type of memory^24–26^, especially in rats using item-in-context paradigms^23,27^, little is known about the underlying neural mechanisms that govern their interactions, and how these learning effects with different temporal characteristics interplay at a network level. In a pivotal experimental task on episodic memory by Panoz-Brown et al.^18^, rats demonstrated high accuracy in solving an item-in-context task, indicating their reliance on episodic memory. Motivated by this item-in-context episodic task, we constructed a computational spiking neural network model to explore the neural mechanisms that govern the intricate interplay between episodic and short-term recency memory effects at a mesoscopic network level. We attributed Panoz-Brown et al.’s^18^ behavioral findings to emergent network dynamics resulting from local synaptic plasticity phenomena operating across various timescales. Our objective was to offer mechanistic insights into these computationally underexplored synergistic memory phenomena. It should be noted that in our computational study we deliberately and consistently referred to the short-term memory phenomena of interest as recency rather than familiarity, used originally by Panoz-Brown et al.^18^. We consider recency a more precise term than familiarity even if the latter has been linked to the general notion of memory strength affected among others by stimulus recency^28^.

### Model predictions and experimental data

Our dual network model successfully matched empirical observations of item-in-context memory in rats (Fig. 1D,E), as reported by Panoz-Brown et al.^18^. Notably, this was achieved while maintaining biologically constrained network connectivity, postsynaptic potential amplitudes, and firing rates compatible with mesoscale recordings from cortex and earlier models. We also generated predictions regarding behavioral outcomes in three modified task paradigms, which could be examined in a follow-up experimental study. In particular, we sought to explore a wider scope of recency effects in episodic memory retrieval.

In our first simulated prediction task we switched the order of odor presentation such that old-in-context items were more recently encoded prior to Memory Assessment than new-in-context items. We then quantified the increase in memory recall performance due to the synergistic contribution of episodic and short-term memory effects of recency. Our findings align with similar experiments in rats that focus on the potential impact of recency on the Object-in-Context task^29^. In that experimental study rats first freely explored an object-A in a visual context-X, and then explored another object-B in context-Y. During a subsequent memory test phase the rats were supposed to choose between object-A or object-B either in context-X or context-Y. The data revealed evidence of enhanced performance when the two items were tested in context-Y (compared to context-X). A contributing factor could be that object-B was more recently encoded than object-A, resulting in a relatively stronger memory trace for object-B compared to object-A at the time of the test. Their reasoning aligns with our mechanistic explanation as we observed strengthened memory traces for recently encoded items (see Fig. 2F).

Our third prediction task with an additional third context could be related to Weisz et al.’s^22^ experiments aimed at examining the impact of memory and time overload on the capacity to recognize new-in-context items. In their experimental protocol rats first freely explored in an open field two non-identical items in different visual contexts labeled as A, B, and C for 5 min each. The subsequent test session took place in one of the arenas (A, B, or C) with two copies of the same object, where only one matched the original spatial location. To receive a reward rats had to identify the new combination of context-object-place with the specific arrangement of the objects. The rats were first tested using two contexts (A, B) and later, in a separate trial, an additional context C was introduced (A, B, C) expanding the complexity of the task. The data shows higher recognition of new-in-context objects for the two context scenario (A, B) compared to the three context scenario (A, B, C) evidencing less recall with increasing requirements. We saw a similar decrease in memory performance in our simulation task, when we increased the number of contexts from two to three.

Our testable predictions may inspire further *behavioral* experiments. We acknowledge that our findings are dependent on the model parameters used in this study to reproduce behavioral findings of an item-in-context memory task in rats^18^. Changes in neuromodulation, noise, external stimuli, task duration and individual variability can influence memory recall in ways not fully captured by our model, though we expect the relative qualitative trends to be preserved.

### A simplified framework for odor-visual system interaction (item-in-context)

The literature supports an object-based framework for odor recollection. Objects (e.g., the smell of strawberry) constitute building blocks of perception and provide the input to visual subsystems for contextual integration. The striking mechanistic similarities between human and rodent data^30^ and between vision, audition, and olfaction^31^ lead us to believe that odors are universally encoded as discrete objects. This object-based encoding aligns well with neural attractor dynamics, which play a crucial role in odor (object) recognition^32,33^, see Methods—Two-network architecture and connectivity). Naturally, in the olfactory system, associative representations in the rodent piriform cortex are densely and reciprocally integrated with limbic and paralimbic areas in the medial temporal lobe and basal forebrain^34^. The interaction between odor and visual information is complex. In our computational approach we relied on a parsimonious model for establishing memory associations between olfactory perception objects, i.e. odor percepts with correlates predominantly in the piriform cortex, and visual objects (e.g., contexts) generated broadly by the visual network system. While these brain systems, comprise distributed network of networks and their inter-connections are also mediated or influenced to some extent by other networks involved in the recollection process (e.g.^34^), we intentionally reduced the model to two long-term-memory-like networks storing odor and visual representations with reciprocal connectivity so that their interactions are in the focus.

Overall, we adopted a parsimonious generic modeling approach for establishing associative memories between items and contexts. These connections may be mediated or influenced by other networks involved in the recollection process, yet we intentionally bypass those additional components to focus on the underlying plasticity mechanisms at the mesoscale level rather than the anatomical aspects of recall.

### Motivation for the learning rule: BCPNN vs. STDP

Our model uses the Bayesian-Hebbian associative learning rule (BCPNN) while there are other associative Hebbian-like learning rules commonly used in computational studies, e.g. spike-timing dependent plasticity (STDP)^35,36^. A key strength of the BCPNN rule lies in the intrinsic regulation of spiking activity through synaptic learning of long-lasting disynaptic inhibition (simulating directly effects of double bouquet cells which may play an important role in shaping neural activity and circuitry^37–40^), and contrasts with potential STDP issues of network stability and robustness in the absence of additional inhibitory circuitry. Indeed, in the absence of disynaptic inhibition our network fails to solve the task as a result of emerging instabilities (Fig. S2). Further, BCPNN integrates Hebbian plasticity with intrinsic excitability in a principled manner also tuned to experimental data^41^, which ensures network stability without requiring additional compensatory mechanisms. Similarly, apart from millisecond time scales of plasticity, comparable to STDP, BCPNN is also equipped with eligibility synaptic traces lasting a few seconds, which facilitate reward-based learning. This allows us to focus on the functional contributions of the learning rule to the episodic memory task.

We do not exclude though that a similar model relying on STDP learning rule that is accompanied by long-range inhibition (e.g. via di-synaptic coupling), intrinsic excitability and eligibility traces mechanisms may explain item-in-context memory. BCPNN offers an elegant framework to encapsulate and tune these effects within a single computational apparatus.

### Immediate recall in episodic memory models

The ability to bind together information within and across episodic events involving temporal ordering (e.g., determining which memory was more recent) is a core cognitive function^42^. Despite the increasing modelling effort to improve our mechanistic understanding of the interaction between temporal dynamics and episodic memory (modelled with long-term Hebbian plasticity) in long time scales^43,44^, little is known about the interactions of recency and processes involved in episodic memories in immediate recall. For example, temporal context models have demonstrated a broad spectrum of recall phenomena in the domain of episodic memory including recency and contiguity effects observed across immediate, delayed, and continuous recall scenarios^45,46^. These models have been proposed to explain temporal-order memory including the idea that episodic memories are encoded with a “temporal context” or “temporally varying signal”, which drifts over time helping to order events^45–47^. In line with our findings, a recent modelling work on newly formed episodic memories, showed that selectively encoding episodic memories at the end of a series of events led to better subsequent prediction performance^48^ due to a reduced interference with earlier presented memories. This conclusion is in line with our study, as we also claim that events encoded at the end benefit immediate episodic recall by enhancing their activations.

Overall, the temporal order-memory and interactions of recency (short-term memory) with episodic memories have been studied, but on longer time scales. Our detailed spiking model represents a novel computational attempt to connect neural and synaptic processes with mesoscopic manifestations underpinning complex effects of short-term memory dynamics on episodic memory in immediate recall. We demonstrated that Bayesian-Hebbian plasticity can explain item-in-context memory and that recency dynamics enhance episodic memory recall by boosting the spiking activity of newly formed episodic memories.

### Generalization over longer time scales

To generalize the model to longer timescales (hours to days), several modifications would be necessary. First, synaptic plasticity parameters—particularly the time constants governing eligibility traces and Hebbian updates—would need to be adapted to support more persistent synaptic changes, possibly by incorporating additional systems-level consolidation processes (e.g., replay, slow-wave activity). It is in the scope of the future work to include such mechanisms and additional network-systems simulating hippocampal contributions that could enhance long-term retention and resistance to interference, however the recency dynamics may not survive such long intervals.

Hippocampus and cortex contribute critically to episodic memory recall, even though our current model implements item-context binding within a cortical network architecture^49,50^. Importantly, the same synaptic plasticity principles we model, for example, the interaction of long‐ and short‐term mechanisms that generate stable attractor dynamics, can operate within hippocampal–cortical networks to form initial episode representations. Over time, however, extensive evidence shows that hippocampal involvement in recall gradually declines as neocortical networks strengthen their own cortico‐cortical bindings through systems consolidation^51,52^. Hippocampal plasticity could be involved in item‐in‐context associations, but the emergent network dynamics, driven by the interplay of plasticity at longer timescales, may enable cortical circuits to sustain long‐term episodic memories independently of hippocampal input.

## Methods

### Neuron model

We use adaptive exponential integrate-and-fire point model neurons, which feature spike frequency adaptation, enriching neural dynamics and spike patterns, especially for the pyramidal cells^53^. This neuron model is an effective model of cortical neuronal activity, reproducing a wide variety of electrophysiological properties, and offers a good phenomenological description of typical neural firing behavior, but it is limited in predicting the precise time course of the subthreshold membrane voltage during and after a spike or the underlying biophysical causes of electrical activity^54^. We slightly modified it for compatibility with the BCPNN synapse model^41^ by integrating an intrinsic excitability current.

Development of the membrane potential *V*_*m*_ and the adaptation current *I*_*w*_ is described by the following equations:

1 $${C}_{m}\frac{d{V}_{m}}{dt}=-{g}_{L}\left({V}_{m}-{E}_{L}\right)+{g}_{L}{\Delta }_{\tau }\frac{{e}^{{V}_{m}-{V}_{t}}}{{\Delta }_{T}}-{I}_{w}\left(t\right)+{{I}_{\beta }(t){+I}_{syn}(t)}+{I}_{ext}$$

2 $$\frac{d{I}_{w}(t)}{dt}=\frac{-{I}_{w}(t)}{{{\tau }_{{I}_{w}}}}+b\delta (t-{t}_{sp})$$

Equation (1) describes the dynamics of the membrane potential *V*_*m*_ including an exponential voltage dependent activation term. A leak current is driven by the leak reversal potential *E*_*L*_ through the conductance *g*_*L*_ over the neural surface with a capacity *C*_*m*_. Additionally, *V*_*t*_ is the spiking threshold, and ∆_T_ shapes the spike slope factor. After spike generation, membrane potential is reset to *V*_*r*_. Spike emission upregulates the adaptation current by *b*, which recovers with time constant *τ*_*Iw*_ (Table 1). To simplify the model, we have removed subthreshold adaptation, which is part of some AdEx models.

Besides a specific external input current *I*_*ext*_, model neurons receive synaptic currents *I*_*synj*_ from conductance-based glutamatergic and GABA-ergic synapses. Glutamatergic synapses feature both AMPA/NMDA receptor gated channels with fast and slow conductance decay dynamics^55,56^, respectively. Current contributions for synapses are described as follows:

3 $${I}_{{syn}_{j}}\left(t\right)={\sum }_{syn} {\sum }_{i} {g}_{ij}^{syn}\left(t\right)\left({{V}_{m}-}{E}_{ij}^{syn}\right)={I}_{j}^{AMPA}(t)+{I}_{j}^{NMDA}(t)+{I}_{j}^{GABA}\left(t\right)$$

### Synapse model and augmentation mechanism

Excitatory AMPA and NMDA synapses have a reversal potential ${E}^{AMPA}={E}^{NMDA},$ while inhibitory synapses drive the membrane potential toward ${E}^{GABA}$. Every presynaptic input spike (at ${t}_{sp}^{i}$ with transmission delay ${t}_{ij}$) evokes a transient synaptic current through a change in synaptic conductance that follows an exponential decay with time constants ${\tau }^{syn}$ depending on the synapse type (${\tau }^{AMPA}\ll {\tau }^{NMDA}$).

4 $${g}_{ij}^{syn}\left(t\right)={{u}_{ij}^{fac}(t){x}_{ij}^{dep}(t){w}_{ij}^{syn}{e}^{\frac{-t-{t}^{i}-{t}_{ij}}{{\tau }^{syn}}}H(t-{t}_{sp}^{i}-{t}_{ij})}$$

$H(\cdot )$ is the Heaviside step function. ${w}_{ij}^{syn}$ is the peak amplitude of the conductance transient, learned by the spike-based BCPNN Learning Rule (next Section). The glutamatergic synapses are also subject to synaptic depression and augmentation with a decay factor *τ*_*D*_ and *τ*_*A*_, respectively (Table 1), following the Tsodyks-Markram formalism^19^, which models the effects of short-term synaptic plasticity^9,10,57^. We chose time-constants from the plausible range of computational fits made on the basis of electrophysiological recordings of cortical pyramidal cells^21^. The utilization factor ${u}_{ij}^{fac}$ represents the fraction of available resources used up by each transmitted spike (a proxy of synaptic release probability), whereas ${x}_{ij}^{dep}$ tracks the fraction of resources that remain available due to transmitter depletion (synaptic depression), with a facilitation time constant ${\tau }_{fac}$ and a depression/reuptake time constant ${\tau }_{rec}$ accordingly:

5 $$\frac{d{u}_{ij}^{fac}}{dt}=\frac{U-{u}_{ij}^{fac}}{{\tau }_{fac}}-U(1-{u}_{ij}^{fac}){\sum } \delta (t-{t}_{sp}^{i}-{t}_{ij})$$

6 $$\frac{d{x}_{ij}^{dep}}{dt}=\frac{1-{x}_{ij}^{dep}}{{\tau }_{rec}}-{u}_{ij}^{fac}{x}_{ij}^{dep}{\sum } \delta (t-{t}_{sp}^{i}-{t}_{ij})$$

**Table 1:** Neuron model and synaptic parameters.

Neuron model parameter	Symbol	Value	BCPNN parameter	Symbol		Value
Adaptation current	b	86 pA	BCPNN AMPA gain	${w}_{gain}^{AMPA}$		0.33 nS
Adaptation decay time constant	${\tau }_{lw}$	280 ms	BCPNN NMDA gain	${w}_{gain}^{NMDA}$		0.03 nS
Membrane capacitance	${C}_{m}$	280 pF	BCPNN bias current gain	${\beta }_{gain}$		40 pA
Leak reversal potential	${E}_{L}$	− 70.6 mV	BCPNN lowest spiking rate	${f}_{min}$		0.2 Hz
Leak conductance	${g}_{L}$	14 nS	BCPNN highest spiking rate	${f}_{max}$		25 Hz
Upstroke slope factor	${\Delta }_{T}$	3 mV	BCPNN lowest probability	*ε*		0.0026
Spike threshold	${V}_{t}$	− 55 mV	P trace time constant	${\tau }_{p}$		30 s
Spike reset potential	${V}_{r}$	− 60 mV	E trace time constant	${\tau }_{e}$		500 ms
Refractory period	${\tau }_{ref}$	5 ms	AMPA Z trace time constant	${\tau }_{Z}^{AMPA}$		5 ms
			NMDA Z trace time constant	${\tau }_{Z}^{NMDA}$		100 ms
			Regular plasticity	${\kappa }_{normal}$		0.3
			Modulated plasticity	${\kappa }_{reward}$		1
Receptor parameter	Symbol	Value	Short-term plasticity parameter	Symbol		Value
AMPA synaptic time constant	${\tau }^{AMPA}$	5 ms	Utilization factor	*U*		0.2
NMDA synaptic time constant	${\tau }^{NMDA}$	100 ms	Augmentation decay time constant	${\tau }_{A}$		5 s
GABA synaptic time constant	${\tau }^{GABA}$	5 ms	Depression decay time constant	${\tau }_{D}$		280 ms
AMPA reversal potential	${E}^{AMPA}$	0 mV				
NMDA reversal potential	${E}^{NMDA}$	0 mV				
GABA reversal potential	${E}^{GABA}$	-75 mV				

### Spike-based BCPNN plasticity

We implement synaptic plasticity of glutamatergic synapses using the BCPNN learning rule^41,58,59^. BCPNN is derived from Bayes rule, assuming a postsynaptic neuron employs some form of probabilistic inference to decide whether to emit a spike or not. Despite that it accounts for the basic Bayesian inference, it is considered more complex than the standard STDP learning rule^60^, and as such it reproduces the main features of STDP plasticity. In a previous study, we demonstrated that with BCPNN synaptic plasticity, but not with standard Hebbian STDP, the model can reproduce traces of semantization as a result of learning^14^. Therefore, in our effort to explore the interplay of episodic memory with recency effects we utilize the BCPNN learning rule.

The BCPNN synapse continuously updates three synaptic biophysically plausible local memory traces, *P*_*i*_, *P*_*j*_ and *P*_*ij*_, implemented as exponentially moving averages (EMAs) of pre-, post- and co-activation, from which the Bayesian bias and weights are calculated. EMAs prioritize recent patterns, so that newly learned patterns gradually replace old memories. Specifically, learning implements exponential filters, Z, E, and P, of spiking activity with a hierarchy of time constants, $\tau$_Z_, $\tau$_E_, and $\tau$_P_, respectively. Due to their temporal integrative nature they are referred to as synaptic (local memory) traces.

To begin with, BCPNN receives a binary sequence of pre- and postsynaptic spiking events (*S*_*i*_, *S*_*j*_) to calculate the traces *Z*_*i*_ and *Z*_*j*_:

7 $${\tau }_{{Z}_{i}}\frac{{dZ}_{i}}{dt}=\frac{{S}_{i}}{{f}_{max}{t}_{spike}}-{Z}_{i}+\varepsilon , {\tau }_{{Z}_{j}}\frac{{dZ}_{j}}{dt}=\frac{{S}_{j}}{{f}_{max}{t}_{spike}}-{Z}_{j}+\varepsilon$$

*f*_*max*_ denotes the maximal neuronal spike rate, *ε* is the lowest attainable probability estimate, *t*_*spike*_ denotes the spike duration while $\tau$_Zi=_$\tau$_Zj_ are the presynaptic and postsynaptic time constants, respectively ($\tau$_Z_ =$\tau$ ^AMPA^ = 5 ms for AMPA, and $\tau$_Z_ =$\tau$^NMDA^ = 100 ms for NMDA components, Table 1).

*E and P* traces are then estimated from the *Z* traces as follows:

8 $${\tau }_{E}\frac{{dE}_{i}}{dt}={Z}_{i}-{E}_{i}, {\tau }_{E}\frac{{dE}_{j}}{dt}={Z}_{j}-{E}_{j}, {\tau }_{E}\frac{{dE}_{ij}}{dt}={Z}_{i}{Z}_{j}-{E}_{ij}$$

9 $${\tau }_{P}\frac{{dP}_{i}}{dt}=\kappa \left({E}_{i}-{P}_{i}\right), {\tau }_{P}\frac{{dP}_{j}}{dt}=\kappa \left({E}_{j}-{P}_{j}\right), {\tau }_{P}\frac{{dP}_{ij}}{dt}=\kappa \left({E}_{i}{E}_{j}-{P}_{ij}\right)$$

The parameter *κ* adjusts the learning rate, reflecting the action of endogenous modulators of learning efficacy (i.e., activation of a D1R-like receptor). Setting *κ* = 0 freezes the network’s weights and biases, though in our simulations the learning rate remains constant (κ_normal_ = 0.3) during encoding. However, to account for the experimental paradigm we trigger a transient increase of plasticity to simulate the impact of a reward signal on the memory system by implementing eligibility traces (see Eq. 8) and upregulating the associative plasticity gain (κ_reward_ = 1) upon successful execution of the task by the model. Each synapse maintains exponentially decaying traces of presynaptic firing, postsynaptic firing, and their co‐occurrence. When the reward arrives after 250 ms, we transiently boost the associative learning gain ${(\kappa )}$ from its baseline (κ_normal_) to an elevated level (κ_reward_). This elevated $\kappa$ converts the accumulated eligibility traces into weight updates, thereby “crediting” the synapses that were active around the decision time despite the delay.

Finally, *P*_*i*_, *P*_*j*_ and *P*_*ij*_ are used to calculate intrinsic excitability *β*_*j*_ and synaptic weights *w*_*ij*_ with a scaling factor *β*_*gain*_ and ${w}_{gain}^{syn}$ respectively (Table 1):

10 $${w}_{ij}^{syn}={w}_{gain}^{syn}log\frac{{P}_{ij}}{{P}_{i}{P}_{j}}, {{I}_{{\beta }_{j}}}={\beta }_{gain}log({P}_{j})$$

BCPNN is a Hebbian-like learning rule, neurons that are coactive are coupled with excitatory connectivity. However, neurons that do not fire together in a certain time window feature low coactivation traces (P_ij_), and based on Eq. (10), the final weight update will produce negative conductance. The negative binding is interpreted as disynaptic inhibition mediated by dendritic targeting regular spiking non-pyramidal (RSNP) cells such as double bouquet cells (DBCs)^37^.

### Two-network architecture and connectivity

**Fig. 6:**
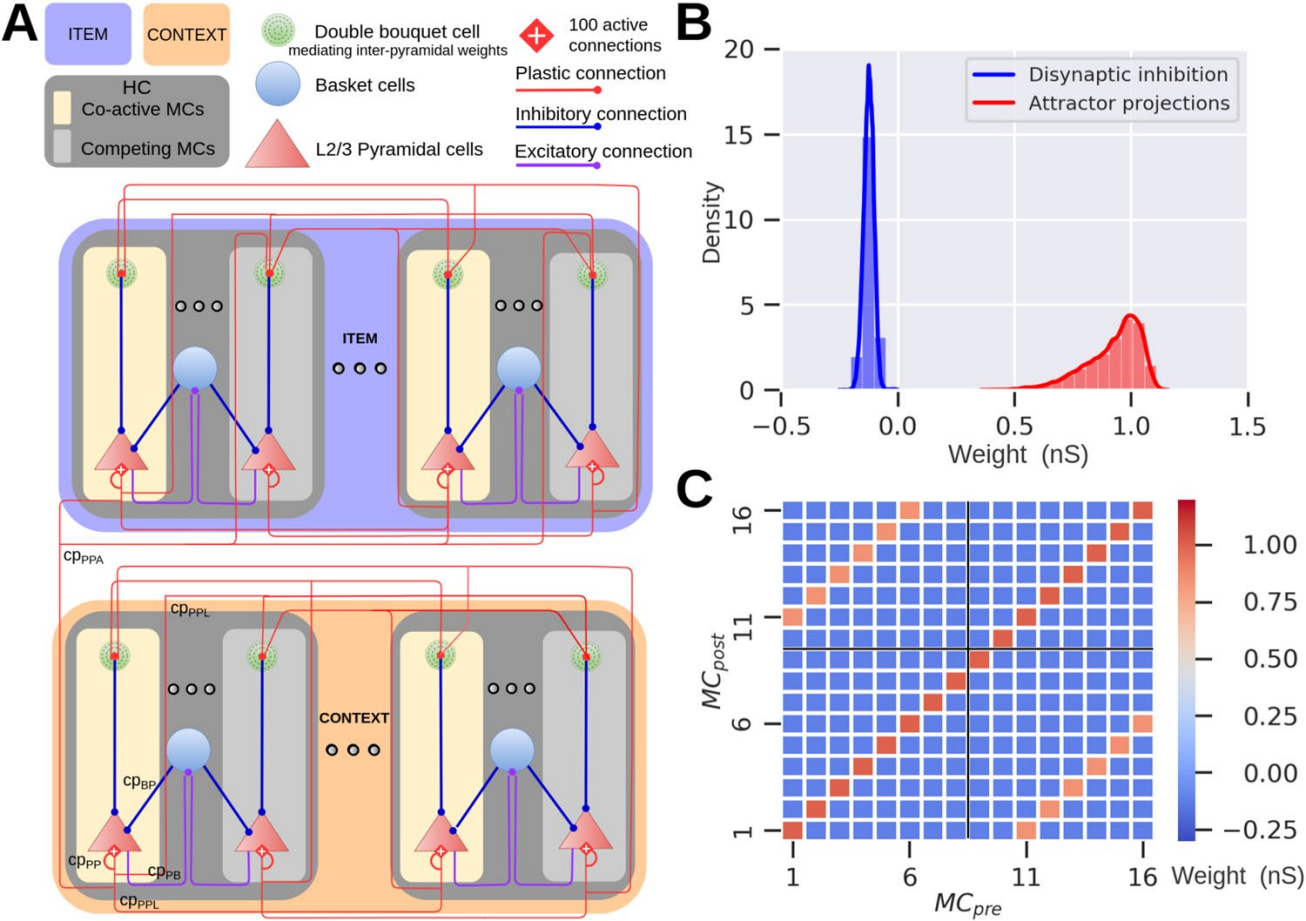
Network architecture and connectivity of the Item (purple-blue) and Context (orange) networks. (**A**) The model represents a subsampled modular cortical layer 2/3 patch consisting of minicolumns (MCs) nested in hypercolumns (HCs). Both networks contain 9 HCs, each comprising 16 MCs. We preload abstract long-term memories of item and context representations into the respective network, in the form of distributed cell assemblies with weights establishing corresponding attractors. Associative plastic connections bind items with contexts. The network features lateral inhibition via basket cells (violet and blue lines) resulting in a soft winner-take-all dynamics. Competition between attractor memories arises from this local feedback inhibition together with disynaptic inhibition between HCs. Unspecific noise and additional specific excitation onto pyramidal cells during stimulation is described in Stimulation Protocol in “Methods”. (**B**) Weight distribution of plastic synapses targeting pyramidal cells. We show the fast AMPA weight components here, but the simulation also includes slower NMDA weight components. (**C**) Weight matrix between attractors and competing MCs across two sampled HCs. The matrix displays the mean of the weight distribution between a presynaptic (*MC*_*pre*_) and postsynaptic minicolumn (*MC*_*post*_), within the same or different HC (black cross separates grid into blocks of HCs, only two of which are shown here). Recurrent attractor connections within the same HC are stronger (main diagonal, dark red) compared to attractor connections between HCs (off-diagonals, orange). Negative pyramidal-pyramidal weights (blue) between competing MCs amounts to disynaptic inhibition mediated by double bouquet cells.

The network model features two reciprocally connected networks, the so-called Item and Context networks. For simplicity, we assume that Item and Context networks are located at a substantial distance accounting for the reduced between-network connection probabilities (Table 2). Each network follows an architecture with modular structure compatible with previous spiking implementations of attractor memory networks^8,9,37,41,61–63^, and is best understood as a subsampled cortical layer 2/3 patch with nested hypercolumns (HCs) and minicolumns (MCs, Fig. 6A). Both networks span a regular spaced grid of 9 HCs (Table 2), each with a diameter of 500 µm^64^. In our model, items are embedded in the Item network and context information in the Context network as internal well consolidated long-term memory representations (cell assemblies), supported with within-network weights (within-network connectivity) derived using prior BCPNN long-term learning (Fig. 6B,C). Consequently, these weights were resistant to changes during associative learning of projections between item and context networks (see “Results” section). Our item and context memory representations are distributed and non-overlapping, i.e. with a single distinct pattern-specific (encoding) MC per HC. This results in a sparse neocortical type of activity patterns^65^. It should be noted that the model tolerates a marginal overlap between different memory patterns, i.e. shared encoding minicolumns (data not shown). Each minicolumn is composed of 30 pyramidal cells (representing the extent of layer 2/3) with shared selectivity, forming a functional (not strictly anatomical) column. In total, the 18 HCs (16 MCs each) of the model contain 8640 excitatory and 1152 inhibitory cells, significantly downsampling the number of MCs per HC (∼100 MCs per HC in biological cortex). Within each HC there are 480 pyramidal cells and 120 basket cells, and hence our model does match in-vivo observations of 4:1 ratio of excitatory to inhibitory cells^66^. Our model also accounts for another type of inhibition—namely, disynaptic inhibition mediated via dendritic targeting double bouquet and/or bipolar cells. As a result, a sizable fraction of the total inhibition (i.e. all the “learned" inhibition) is modeled implicitly via learned negative weights rather than explicitly via inhibitory cells. The high degree of recurrent connectivity within^67,68^ and between MCs links coactive MCs into larger cell assemblies^69–72^. Long-range bidirectional between-network connections (item-context bindings or associative connections) are plastic (shown in Fig. 6A only for MC1 in HC1 of the Context network), binding items and contextual information (Ranganath, 2010). Even though we coupled odor items with contextual information in rapid succession and directly tested recollection in line with the experimental task (Fig. 1B), we used longer time constants for the Hebbian plasticity implementing an intermediate-term episodic binding, and shorter time constants for other coexisting plasticity mechanisms with short-lived effects (e.g., recency). On average, recurrent connectivity establishes 100 active plastic synapses onto each pyramidal cell from other pyramidals with the same selectivity, due to a sparse between-network connectivity (*cp*_*PPA*_) and denser local connectivity (*cp*_*PP*_, *cp*_*PPL*_; connection probability refers to the probability that there is a connection between a randomly selected pair of neurons from given populations; in Fig. 6A connection probabilities are only shown for MC1 in HC1 of the Context network). The model yields biologically plausible excitatory postsynaptic potentials (EPSPs) for connections within HCs (0.72 ± 0.085 mV), measured at resting potential *E*_*L*_^67^. Densely recurrent non-specific monosynaptic feedback inhibition mediated by fast spiking inhibitory cells (Kirkcaldie, 2012) implements a local winner-take-all structure^69^ amongst the functional columns. Inhibitory postsynaptic potentials (IPSPs) have an amplitude of − 1.160 mV (± 0.003) measured at − 60 mV^67^. These bidirectional connections between basket and pyramidal cells within the local HCs are drawn with a 70% connection probability. Notably, double bouquet cells shown in Fig. 6A, are not explicitly simulated, but their effect is nonetheless expressed by the BCPNN rule. A recent study based on a similar single-network architecture (i.e. with the same modular organization, microcircuitry, conductance-based AdEx neuron model, cell count per MC and HC) demonstrated that learned mono-synaptic inhibition between competing attractors is functionally equivalent to the disynaptic inhibition mediated by double bouquet and basket cells^37^. Therefore, BCPNN describes the effect of not-explicitly simulated double-bouquet cells (DBCs) by replacing disynaptic inhibition with negative connections (GABA reversal potential) between cell assemblies that do not share the same pattern selectivity. Also, other network models with negative synaptic weights have been shown to be functionally equivalent to ones with both excitatory and inhibitory neurons with only positive weights^73^. Parameters characterizing other neural and synaptic properties including BCPNN can be found in Table 1.

**Table 2:** Network layout, connectivity and stimulation protocol.

Layout	Symbol	Value	Connectivity	Symbol	Value	Stimulation	Symbol	Value
Cortical patch size	${C}_{ps}$	1.5 × 1.5 mm	Axonal conduction speed	*V*	0.2 m/s	Background noise PYR	${r}_{bg}^{PYR}$	470 Hz
Simulated HCs (each network)	${n}_{HC}$	9	Myelinated axonal speed	${V}_{myel}$	2 m/s	Background noise BA	${r}_{bg}^{BA}$	0 Hz
Simulated MCs (each network)	${n}_{MC}$	144	Minimal synaptic delay	${t}_{min}^{syn}$	1.5 ms	Background conductance	$g{r}_{bg}^{PYR}$	${\pm }1.5 nS$
Simulated MCs per HC	${n}_{MC}^{HC}$	16	Hypercolumn diameter	${d}_{HC}$	0.5 mm	Stimulation duration	${t}_{stim}$	250 ms
No. of items	${n}_{ITEM}$	16 (from 16)	Distance between networks	${d}_{CONTEXT}^{ITEM}$	10 mm	Stimulation rate	${r}_{stim}$	340 Hz
No. of contexts	${n}_{CONTEXT}$	2 (from 16)	PYR-PYR recurrent cp	${cp}_{PP}$	0.2	Stimulation before reward	${t}_{cue}$	100 ms
Layer 2/3 pyramidal per MC	${n}_{MC}^{PYR-L23}$	30	PYR-PYR long-range cp	${cp}_{PPL}$	0.2	Stimulation conductance	${g}_{stim}$	${+}$ 1.5 nS
Basket cells per MC	${n}_{MC}^{Basket}$	4	PYR-PYR associative cp	${cp}_{PPA}$	0.04	Interstimulus interval	${T}_{stim}$	200 ms
MC grid size (Item + Context)	${G}_{MC}^{TOTAL}$	18 × 16	PYR-BA cp, BA-PYR cp	${cp}_{PB},{cp}_{BP}$	0.7			
			PYR-BA cc	${g}_{PB}$	3 nS			
			BA-PYR cc	${g}_{BP}$	-7 nS			

PYR, Pyramidal cell; BA, Basket cell, cp, connection probability; cc, connection conductance.

Figure 6B shows the weight distributions of embedded distributed cell assemblies, representing different memories stored in the Item and Context networks. Attractor projections can be further categorized into strong local recurrent connectivity within HCs, and slightly weaker long-range excitatory projections across HCs (Fig. 6C).

### Axonal conduction delays

Conduction delays (*t*_*ij*_) between a presynaptic neuron *i* and a postsynaptic neuron *j* are calculated based on their Euclidean distance, *d*, and a conduction velocity *V* (Eq. 11). Delays are randomly drawn from a normal distribution with a mean according to distance and conduction velocity, with a relative SD of 30% of the mean in order to account for individual arborization differences, and varying conduction speed as a result of axonal thickness and myelination. In addition, a minimal delay of 1.5 ms (${t}_{min}^{syn}$, Table 2) is added to reflect synaptic delays due to effects that are not explicitly modeled, e.g. diffusion of neurotransmitters over the synaptic cleft, dendritic branching, thickness of the cortical sheet and the spatial extent of columns^67^. Associative between-network projections have a ten-fold faster conduction speed than those within each network, reflecting axonal myelination.

11 $$\overline{{t}_{ij}}= \frac{\sqrt{{\left({x}_{i}-{x}_{j}\right)}^{2}+{\left({y}_{i}-{y}_{j}\right)}^{2}}}{V}+{t}_{min}^{syn}, {t}_{ij} \sim N(\overline{{t}_{ij}} , .3\overline{{t}_{ij}})$$

### Stimulation protocol

Noise input to pyramidal cells is a zero-mean noise, generated by two independent Poisson generators with opposing driving potentials. Pyramidal cells coding for specific items and contexts are stimulated with an additional specific excitation during encoding and cued recall (all parameters in Table 2).

### New- vs. old-in-context memory discrimination

Our model discriminates old- vs. new-in-context items based on a comparison of the firing rates in the Item network during stimulation of items in given contexts. New-in-context items were selected if the corresponding trial-average firing rates were 15% lower than the pair-matched old-in-context items. First, we determined a decision threshold high enough to show significant differences between trial-average firing rates, and then we tuned the model (i.e., strength of activations-cues and background excitation—noise) to match the reported behavioral results of an item-in-context memory task. By changing this decision threshold, we can retune the strength of the cues and noise, or even modify other parameters (i.e., boost between-network connectivity) to produce comparable results, so the decision threshold by itself is not critical. The model is also robust to changes in cue strength, slightly weaker and stronger cues still allow reliable discrimination of old-in-context items, as their activation is strongly influenced by the simultaneously cued context through the learned item-context binding. Contextual input helps disambiguate memory representations (boosting old-in-context item activation while suppressing new-in-context item activation). Thus, the model is capable of preserving the reported behavior and trends, for example, effect of recency between Arrangement 1 vs. Arrangement 2 (Fig. 2A,B). The exact performance metrics may be subject to changes, however, synaptic model parameters can be tuned to counteract any potential changes e.g., upregulating or downregulating synaptic gain to boost or reduce the influence of context onto old- and new-in-context items. Similar effects can be observed with the decision threshold—lowering or increasing the decision threshold preserves trends in the observed modelling predictions of our study, while potential differences in the performance scores can be alleviated by tuning synaptic model parameters to regulate behavioral manifestations imposed by changes in the decision threshold. It is worth mentioning that, we did observe that increasing unspecific background noise beyond a certain threshold leads to noise-driven activations which disrupt cued-drive activations, i.e., spontaneous memory reactivations not triggered by external cues. To avoid this, we operated in a regime where background noise is sufficient to facilitate cue-driven activation (by keeping the network near a responsive state) but not strong enough to induce uncontrolled, spontaneous attractor state transitions.

We interpreted the firing rates of the cell assemblies as a proxy for downstream readout in order to isolate and examine the interactions of synaptic plasticity mechanisms during encoding and recall. Lower firing of the new-in-context assembly was interpreted as reduced drive onto a downstream decision layer of inhibitory networks, thereby disinhibiting the action channel that selects the “new” response. While the action selection following the recollection (old item-in-context) is an intriguing subject, it also fell out of the scope of this particular study to detail it further.

### Pairwise differences

To show changes in firing rates (f), within- and between-network connectivity (w), and bias (b), we calculate the corresponding differences in the averages between pairs of old- and new-in-context items, Δf_old-new_, Δw_old-new_, Δb_old-new_, respectively.

## Supplementary Information

Supplementary Information.

## Data Availability

We performed model simulations using the NEST simulator^74^ version 2.2.2, and a custom-built Bayesian–Hebbian learning rule module (BCPNN^41^) in NEST, running on an HPE Cray EX supercomputer. The BCPNN learning rule, model simulation code and materials used to generate the results in thiswork are available on github (https://github.com/Nikolaos-Chrysanthidis/Short-term-plasticity-influences-episodic-memory-recall). An earlier version of themodel is also available on ModelDB (https://modeldb.science/257610). Further requests for materials should be directed to the corresponding author.
