# Supplementary material for: Short-term plasticity influences episodic memory recall: an interplay of synaptic traces in a spiking neural network model
Source: Sci Rep. 2025 Aug 1;15:28164. doi: 10.1038/s41598-025-12611-5 (PMC12316876; doi:10.1038/s41598-025-12611-5)
Supplement: Supplementary file 1 — Supplementary Information. [file 41598_2025_12611_MOESM1_ESM.docx]

**Supplementary Material**

**
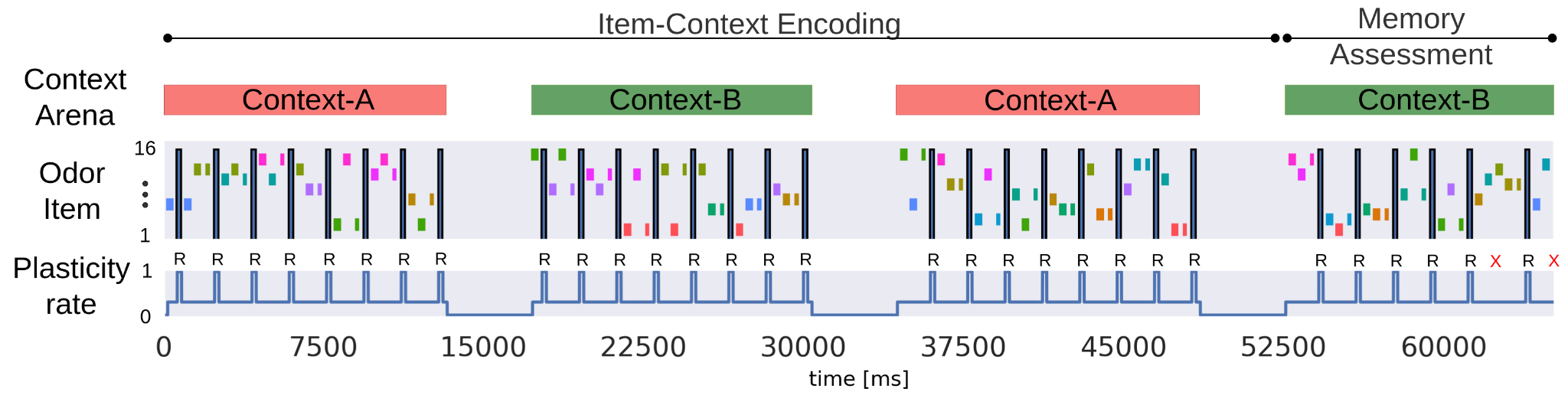
**

**Figure S1**: Graphical schematic of the three-context-transition task displaying pairs of new-old odors (depicted as rectangles with unique colors) in a given context. Odors were presented across two contexts in the simulated episodic memory task, and only the new-in-context items were rewarded (**R** symbol in the schematic denotes reward, and **X** symbol, in red, indicates a failed trial) when selected (a 50 ms stimulation of the selected odor preceded the reward phase, representing a final odor sniff before the reward). Once a new-in-context item was presented it was considered as old-in-context for the subsequent trials in the given context (as a trial we defined a stimulation of a pair of new- and old-in-context items). Items were stimulated for the first time in context-A, half of the total 16 items were presented and rewarded in context-A. After the context transition half of the 16 items were presented in random pairs in context-B. After one more context transition, we activated in context-A the remaining 8 items that were not previously presented in that context. Finally, Memory Assessment was made in context-B, where we presented the remaining half of the items that had not been presented in context-B, and paired them randomly with old-in-context-B items (pairs of odors were different throughout the task). Context representations were constantly activated while cueing pairs of new-old items for 250 ms each. In the Memory Assessment block, pairs of new-old items followed the Arrangement 1 criterion (new-in-context-B items were encoded more recently in the preceding context than the old-in-context-B ones). While context representations were persistently cued we activated new- and old-in-context items during trials. Plasticity rate of the associative binding between Item and Context networks was modulated during item presentation and rewarded accordingly (bottom subplot).

**
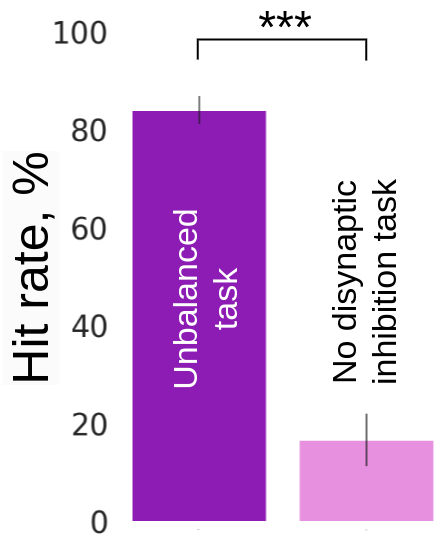
**

**Figure S2**: Average recall performance (hit rate, %) for the unbalanced and “No disynaptic inhibition” prediction tasks corresponding to Arrangement 1 configuration. For the “No disynaptic inhibition” task, the inhibitory weights between networks were disabled, and thus the Context network did not suppress the new-in-context-A items during Memory Assessment. SDs derived from the Bernoulli distributions for the probabilities of success (hit) across all trials (scaled to %).
